# Genetic population structure and relatedness in the narrow‐striped mongoose (*Mungotictis decemlineata*), a social Malagasy carnivore with sexual segregation

**DOI:** 10.1002/ece3.2123

**Published:** 2016-05-05

**Authors:** Tilman C. Schneider, Peter M. Kappeler, Luca Pozzi

**Affiliations:** ^1^ Department of Sociobiology/Anthropology University of Göttingen Kellnerweg 6 D‐37077 Göttingen Germany; ^2^ Behavioral Ecology & Sociobiology Unit German Primate Center Leibniz Institute for Primate Research Kellnerweg 4 D‐37077 Göttingen Germany

**Keywords:** Eupleridae, male associations, matrilines, mongoose, *Mungotictis decemlineata*, reproductive skew

## Abstract

Information on the genetic structure of animal populations can allow inferences about mechanisms shaping their social organization, dispersal, and mating system. The mongooses (Herpestidae) include some of the best‐studied mammalian systems in this respect, but much less is known about their closest relatives, the Malagasy carnivores (Eupleridae), even though some of them exhibit unusual association patterns. We investigated the genetic structure of the Malagasy narrow‐striped mongoose (*Mungotictis decemlineata*), a small forest‐dwelling gregarious carnivore exhibiting sexual segregation. Based on mtDNA and microsatellite analyses, we determined population‐wide haplotype structure and sex‐specific and within‐group relatedness. Furthermore, we analyzed parentage and sibship relationships and the level of reproductive skew. We found a matrilinear population structure, with several neighboring female units sharing identical haplotypes. Within‐group female relatedness was significantly higher than expected by chance in the majority of units. Haplotype diversity of males was significantly higher than in females, indicating male‐biased dispersal. Relatedness within the majority of male associations did not differ from random, not proving any kin‐directed benefits of male sociality in this case. We found indications for a mildly promiscuous mating system without monopolization of females by males, and low levels of reproductive skew in both sexes based on parentages of emergent young. Low relatedness within breeding pairs confirmed immigration by males and suggested similarities with patterns in social mongooses, providing a starting point for further investigations of mate choice and female control of reproduction and the connected behavioral mechanisms. Our study contributes to the understanding of the determinants of male sociality in carnivores as well as the mechanisms of female competition in species with small social units.

## Introduction

The social organization of vertebrates is defined by the composition of species‐specific social units with regard to the number of individuals, their sex, age, cohesion, and the resulting genetic structure (Kappeler and van Schaik 2002). Behavioral and demographic data can be used to describe how individuals are distributed in space and time, and solitary, pair‐living, and group‐living species are recognized as fundamental units of categorization of interspecific variation in social organization (Crook 1970). The resulting genetic structure of a given population and, in particular, the relatedness within and among social groups is not straightforward to predict from a particular type of social organization, however, because species vary in sex‐specific reproductive skew and dispersal pattern, adult sex ratio and other demographic characteristics (Greenwood 1980; Nonacs and Hager 2011; Di Fiore 2012; Liker et al. 2013; Nidiffer and Cortés‐Ortiz 2015). Information on genetic structure can therefore reveal insights about social units, population structure, and social behavior that are not obvious from behavioral and demographic data alone (Gompper et al. 1997; Kappeler et al. 2002; Caniglia et al. 2014). As sex differences in reproductive success affect the balance between local resource competition and local mate competition (Perrin and Mazalov 2000), information on local genetic population structure also allows characterization of these components of the mating system (Ross 2001).

Among mammals, the Carnivora exhibit fascinating interspecific diversity in social organization and breeding systems (Macdonald 1983; Bekoff et al. 1984). Molecular studies have contributed to the elucidation of social organization and reproductive patterns of some enigmatic carnivores such as lions, *Panthera leo*, cheetahs, *Acinonyx jubatus*, and spotted hyenas, *Crocuta crocuta* (Packer et al. 1991; Gottelli et al. 2007; Holekamp et al. 2012). In several species, reproductive skew within social units has been estimated explicitly, tackling diverse questions about the extent of breeding competition, mate choice, kin selection among competitors, and alternative reproductive strategies (e.g., African wild dogs, *Lycaon pictus*: Girman et al. 1997; badgers, *Meles meles*: Dugdale et al. 2008; Ethiopian wolves, *Canis simensis*: Randall et al. 2007; lions: Packer et al. 1991, 2001; spotted hyenas: Engh et al. 2002; East et al. 2003; white‐nosed coatis, *Nasua narica*: Gompper et al. 1997). Much less is known about genetic consequences of social organization and mating system in solitary and forest‐dwelling carnivores, however, because they are typically elusive, wide‐ranging, and have high dispersal capabilities (e.g., Dutta et al. 2013; Rodgers et al. 2015).

The sister taxa Herpestidae (mongooses) and Eupleridae (Malagasy carnivores, including the Galidiinae, Malagasy mongooses) are socially and ecologically diverse groups of carnivores (Schneider and Kappeler 2014). Genetic studies of social herpestids have focused on breeding success and patterns of inbreeding (Keane et al. 1996; Griffin et al. 2003; Nielsen et al. 2012; Leclaire et al. 2013). Insights into their social and genetic mating systems have contributed significantly to the development and testing of reproductive skew models (Cant et al. 2001, 2010; Clutton‐Brock et al. 2001a, 2008). Recent studies on banded mongooses, *Mungos mungo*, have revealed important information on spatiotemporal population‐wide genetic variation and within‐group relatedness. They identified the genetic determinants of the mating system and social behaviors, and particularly the links between extra‐group paternity, mate choice, and female control of paternity on the one hand, and inbreeding on the other hand (Nichols et al. 2012, 2015; Sanderson et al. 2015). In contrast, much less is known about genetic structure and reproductive systems of the so‐called “solitary” mongooses, which often display gregarious tendencies (e.g., *Galerella sanguinea*: Waser et al. 1994). For the Malagasy carnivores, information on local genetic population structure is missing, apart from relatedness estimations of male dyads in fossa, *Cryptoprocta ferox* (Lührs et al. 2013; Schneider and Kappeler 2014). Existing molecular studies in euplerids have focused on a higher organizational level, addressing phylogeographic and taxonomic questions (Veron et al. 2004; Bennett et al. 2009; Durbin et al. 2010; van Vuuren et al. 2012). Since they have evolved in isolation on Madagascar for more than 20 myr (Yoder et al. 2003), potential convergences with African herpestids are particularly interesting for behavioral ecologists.

Genetic structure can differ between the maternally inherited mitochondrial and the diploid inherited nuclear DNA, even on small geographic scales. Difference in mitochondrial DNA can be maintained between social groups in spite of nuclear gene flow (Avise et al. 1987; Hoelzer et al. 1994). Therefore, the combined use of mtDNA and nuclear microsatellites has been worthwhile to detect sex‐specific genetic structure and different processes acting on evolutionary and ecological timescales (Rassmann et al. 1997; Gerloff et al. 1999; Haavie et al. 2000; Martien et al. 2014).

**Figure 1:**
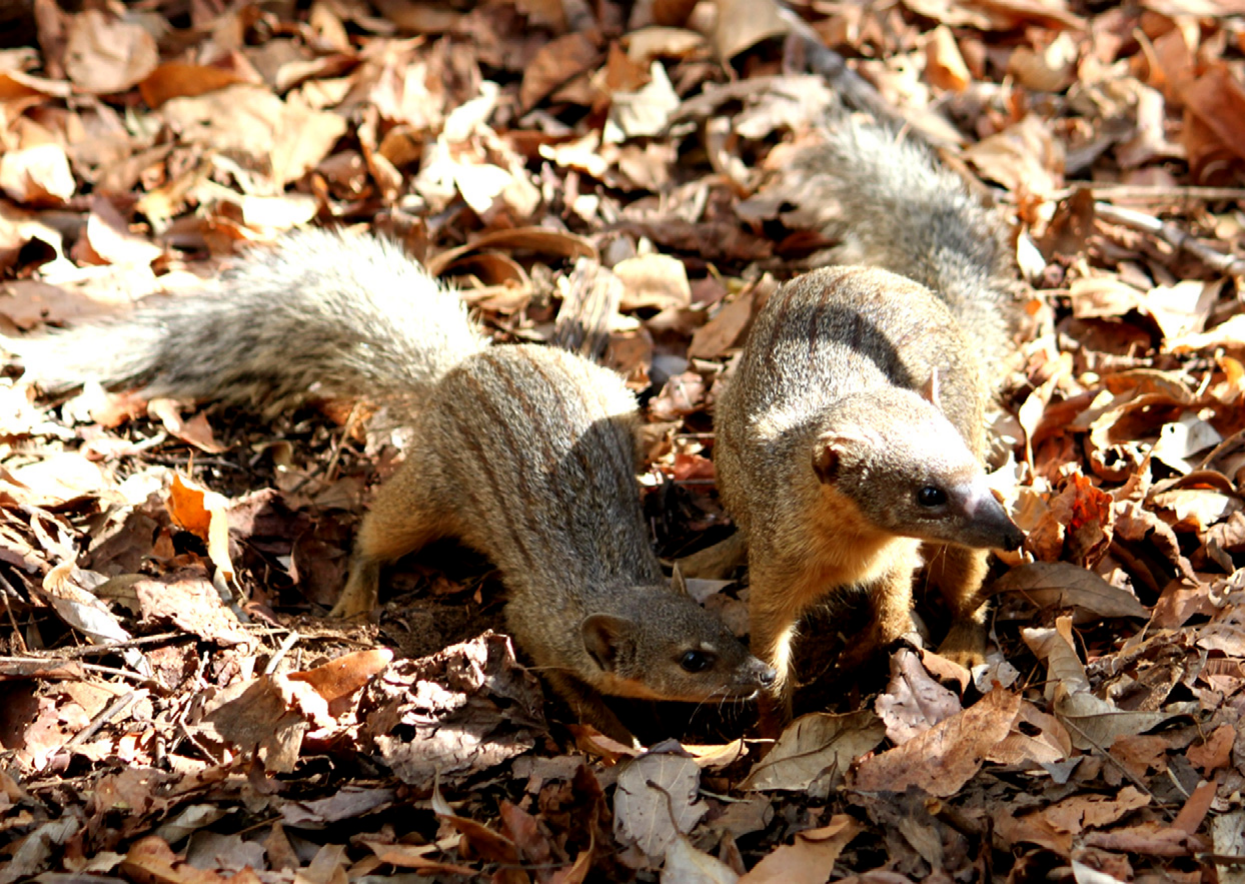
Adult female and juvenile of *Mungotictis decemlineata*.

In the present study, we characterize the local population genetic structure, relatedness, and reproductive system of the Malagasy narrow‐striped mongoose, *Mungotictis decemlineata* (Fig. 1). The social organization of this small diurnal, forest‐dwelling, largely insectivorous carnivore has only recently been described (Schneider and Kappeler, in press) and revealed unique social patterns. Spatial and behavioral data of 40 radiocollared individuals collected over 4 years revealed that 1–3 adult females and their offspring form stable social units that occupy exclusive home ranges. Adult males form separate social units, which also contain 2–4 individuals, but which cover the home ranges of on average three female social units and which disintegrate during the annual mating season. The adult sex ratio and natal dispersal are male‐biased, while females often remain and breed in their natal units. Reproduction is seasonal. Females give birth to only one young by the end of the dry season (Oct–Dec). In cases in which females lose their pup, they can become receptive again and give birth approximately 3 months later (February–March). According to our observations, only the pup of the oldest and dominant female of each unit survives the first year of life. Pup care is generally provided by the mother, without evidence of cooperative breeding in the sense of allosuckling and pup feeding (Schneider and Kappeler, in press).

The goal of this study was to glean additional information on the genetic basis of this unusual type of social organization. Specifically, we aimed to clarify whether (1) there are population‐wide sex differences in haplotype distribution and relatedness, (2) the higher observed rate of male dispersal was reflected by greater haplotype diversity, (3) members of male and female social units were more closely related to each other than expected by chance, and (4) to characterize the genetic mating system and the level of reproductive skew in both sexes. By providing more details on the genetic structure and reproductive system of this representative of a largely unstudied group of carnivores, we also aimed to derive determinants of this type of social organization and thereby contribute to the understanding of the evolutionary pathways from solitariness to gregariousness.

## Materials and Methods

### Study site and data collection

The study was conducted in Kirindy Forest/CNFEREF (44°39′E, 20°03′S), a dry deciduous forest of about 12,500 ha located 60 km northeast of Morondava in western Madagascar. The region is characterized by pronounced seasonality with a cool dry season between May and October and a hot rainy season with an average of 900 mm of annual precipitation (Sorg and Rohner 1996; Kappeler and Fichtel 2012). Narrow‐striped mongooses were regularly trapped in two local study areas about 2 km apart. Morphometric, behavioral, life‐history, and spatial data were collected from radiocollared individuals of nine female units (A–J) and 13 (#1–13) male associations on a daily basis during regular census (including identity, sex, age, number of associates and location, behavioral interactions) and continuous tracking year‐round between 2011 and 2014 (Schneider and Kappeler, in press). For the present study, we obtained small skin biopsies from 96 anaesthetized individuals, including 23 samples collected during a previous capture study between 2000 and 2010, for which data on social units are not available. No negative effects of anesthesia or sampling procedures were observed. All applicable international, national, and institutional guidelines for the care and use of animals were followed. Research protocols, capture procedures, and sample export were approved and permitted by the Ministry for the Environment, Water and Forests of Madagascar, MINEEF. We transferred the samples in 90% ethanol to the genetics laboratory of the German Primate Center.

### Genetic analyses

#### mtDNA

DNA for all analyses was extracted from the tissue samples of all 96 captured individuals using the Qiagen QIAamp Tissue Kit (Qiagen, Hilden, Germany). We amplified a fragment of the mtDNA control region (d‐loop) via PCR, using the mammalian primers ProL‐He (5′‐ATACTCCTACCATCAACACCCAAAG‐3′) and DLH‐He (5′‐GTCCTGAAGAAAGAACCAGATGTC‐3′; Seddon et al. 2001). In a 30 *μ*L reaction, 2 *μ*L DNA extract (50 ng), 18.4 *μ*L H_2_O, 3 *μ*L 10× buffer (containing MgCl_2_ 15 mmol/L), 0.1 *μ*L of each primer (100 pmol/*μ*L), 0.2 *μ*L dNTP (25 mmol/L), 4.0 *μ*L BT (10 mg bovine serum albumin [BSA] + 0.5% Triton), and 0.2 *μ*L BioTherm™ Taq DNA polymerase (5 units/*μ*L, GeneCraft^®^, Ares Bioscience GmbH, Köln, Germany) were used. Cycling conditions in Thermocycler 2720 (Applied Biosystems^®^, Thermo Fisher Scientific, Waltham, MA, U.S.A.) were as follows: an initial step of 92°C for 10 min, followed by 40 cycles of 1 min at 92°C, 1 min at 60°C, and 1 min at 72°C, followed by an elongation step of 5 min at 72°C. Amplification success was validated by visualization of PCR products under UV light (312 nm) after electrophoresis on a 1% agarose gel (Gelred™; Biotium Inc., Hayward, CA).

To reduce the chance of sequencing mitochondrial pseudogenes in the nuclear genome (numts), we also amplified 31 (37%) of the sequences by long‐template PCR. We targeted a region of about 2kbp using a newly designed primer, L14724C (CGACTAATGATATGAAAAACCATCGTTG; modified from Irwin et al. 1991), and one of the primers reported above, DLH‐He. Results from LR‐PCRs and short fragment amplifications were then compared to validate our approach. PCR products were purified using Microcon^®^ UFC7 PCR Centri filters (Merck Millipore, Billerica, MA). For sequencing, in a 10 *μ*L reaction, 6.5 *μ*L purified PCR product (20 ng), 1.5 *μ*L 5× BigDye^®^ buffer, 1.0 *μ*L primer (3.3. pmol/*μ*L), and 1.0 *μ*L Big Dye^®^ were used. PCR products were precipitated by a natrium–acetate–ethanol mix and sequenced on an ABI 3130xL Genetic Analyzer (Applied Biosystems^®^).

Chromatograms were checked by eye, and consensus for each individual was generated from sequences in forward and reverse directions using Geneious R6.1.4 (Biomatters Ltd, Auckland, New Zealand). We aligned the final sequences using MUSCLE (Edgar 2004) and estimated mitochondrial genetic diversity including total number of haplotypes, haplotype diversity (Hd), and nucleotide diversity (*π*) using DNaSP 5.10.1 (Librado and Rozas 2009). In order to assess haplotype and nucleotide diversity between males and females, we tested for significance using the “difference test” in Statistica ver. 9.1 (StatSoft Inc. 2010). Finally, we created a minimum spanning network (Bandelt et al. 1999) using PopArt ver. 1.7 (Leigh and Bryant 2015).

The control region of *M. decemlineata*, as in many other carnivore species, is characterized by multiple repetitive sequences (RS2 and RS3, Hassanin and Veron 2016). As a consequence, amplicons in our study varied in length due to the presence of indels across samples. We performed long‐range PCRs on multiple samples to minimize the risk of including numts in our analyses. Long‐ and short‐template PCRs showed identical sequences; therefore, we are confident that our dataset contains only true mitochondrial sequences. Given the difficulty of dealing with gaps in estimating mitochondrial haplotypes, we employed a conservative approach in which gap data due to the presence of indels were excluded from the analyses (as implemented in PopArt ver. 1.7; Leigh and Bryant 2015). The final alignment included 325 bp. MtDNA sequences were uploaded to GenBank and can be accessed through the following accession numbers KU696553–KU696636.

#### Amplification and analyses of microsatellites

For this analysis, we isolated genomic DNA from all 96 individuals. We tested 26 microsatellite markers isolated for the fossa (*Cryptoprocta ferox)* to characterize genetic variation (Vogler et al. 2009). Thirteen markers were polymorphic with an average number of eight alleles (range: 3–12; Table S1). One of each primer pair was end‐labeled with a FAM‐fluorescent tag. In a 30 *μ*L reaction, 2 *μ*L DNA extract (50 ng), 20.4 *μ*L H_2_O, 3 *μ*L 10× buffer (containing MgCl_2_ 15 mmol/L), 0.1 *μ*L of each primer (100 pmol/*μ*L), 0.2 *μ*L dNTP (25 mmol/L), 4.0 *μ*L BT (10 mg bovine serum albumin [BSA] + 0.5% Triton), and 0.2 *μ*L BioTherm™ Taq DNA polymerase (5 units/*μ*L) were used. The amplification was carried out as follows: 92°C for 10 min, 35 cycles of denaturation at 92°C for 30 sec, annealing at primer‐specific temperature (Table S1) for 30 sec, extension at 72°C for 30 sec; final extension step at 72°C for 10 min. Microsatellites were analyzed on an ABI 3130xL Genetic Analyzer (Applied Biosystems^®^). Fragment length was assessed relative to GeneScan™‐400HD Size Standard using peak scanner™ v1.0 (Applied Biosystems^®^).

We tested for deviations from Hardy–Weinberg equilibrium (HWE) and linkage disequilibrium (LD) using the package “pegas” ver. 0.7 (Paradis 2010) in R 3.2.1 (R Core Team 2015). For HWE, *chi‐square* tests and for LD, the *T_2_* test (Zaykin et al. 2008) were implemented in this package. *F*_*IS*_ estimates according to Weir and Cockerham (1984) and Robertson and Hill (1984) were calculated in GENEPOP ver. 4.3. (Raymond and Rousset 1995; Rousset 2008). We conducted estimations of expected and observed homozygotes and tests for the presence of null alleles using MICRO‐CHECKER (Van Oosterhout et al. 2004). Three of 13 loci showed significant deviations from HWE and signs of null alleles (loci 41HDZ71, 41HDZ105, and 41HDZ626; Table S2). For the following analyses of relatedness and parentages, we used 12 loci; locus 41HDZ71 was excluded due to many null alleles (up to 41%; Table S2). The test for LD did not reveal nonrandom associations of alleles among different loci (*T*_2_ = 23.97; *df *= 22; *P *=* *0.349).

#### Relatedness analyses based on microsatellite data

Since field observations on social unit composition and maternities were not available from the period before 2010, we conducted parentage and relatedness analyses only for the 73 individuals sampled between 2010 and 2014, based on the 12 selected appropriate loci (see above). Several methods to estimate relatedness among individuals have been proposed in the literature; however, their performance is influenced by many factors, including the characteristics of the molecular markers used (e.g., number of loci, heterozygosity, allele frequency) and the pedigree of the individuals being examined (Csilléry et al. 2006; Pew et al. 2015). In order to select the best relatedness estimator based on our data, we used the R package *related* (Pew et al. 2015). This package implements the functionality of COANCESTRY (Wang 2011), but it also allows for an explicit comparisons across seven different relatedness estimators, five moment estimators (Queller and Goodnight 1989; Li et al. 1993; Ritland 1996; Lynch and Ritland 1999; Wang 2002) and two likelihood‐based estimators, the dyadic likelihood estimator – dyadml (Milligan 2003) and the triadic likelihood estimator – trioml (Wang 2007). Using the allele frequencies observed in our dataset, we first simulated datasets of 100 pairs for four known relatedness categories (parent–offspring, full‐sibling, half‐sibling, and unrelated). Then, for each of the seven relatedness estimators, we calculated the correlation coefficient (“Pearson's”) between observed and expected relatedness values.

We then calculated the average relatedness within categories at three different levels: (1) adult males versus adult females; (2) among adult females within the same social unit (eight units with 2 or more adult females); and (3) among males belonging to the same male association (11 associations with at least two genotyped individuals). To determine whether individuals within categories were more closely related than expected, we compared the observed values for each category against a distribution of “expected” relatedness values generated by randomly shuffling individuals between categories groups for 1000 permutations and keeping size constant using the R package *related* (Pew et al. 2015). If the observed mean relatedness was greater than that of the permuted data (*P *>* *0.95), then the null hypothesis, that the mean within‐category relatedness is random, was rejected.

#### Parentage and sibship analyses

We used COLONY V2 (Jones and Wang 2010) to infer parentages and full‐ and half‐sibship, based on 12 microsatellite loci. COLONY is robust against deviations from HWE (J. Wang, pers. comm.). Therefore, two of the loci showing deviations from HWE and marginal signs of null alleles (41HDZ105 and 41HDZ626) remained in the analysis. However, we set a higher genotyping error rate of 0.03 for these two loci (J. Wang, pers. comm.). For all other loci, a genotyping error rate of 0.01 was set, according to estimations based on a number of resampled individuals (c. 7% of the study population; Hoffman and Amos 2005). We implemented the recommended settings of full‐likelihood method and a polygamous mating system without inbreeding (appropriate when analyzing parentages for offspring from several breeding seasons; Jones and Wang 2010). The probability that fathers and mothers were included in the candidate samples was assumed to be 80% for males and 90% for females, based on the mean proportion of sampled individuals of all marked and unmarked individuals per year. Based on the seasonal reproduction, cohorts included animals born at the end (October–December) of the respective year or at the beginning (February–March) of the following year. The offspring sample included the four cohorts 2010–2013. Four females of the cohort 2010 were included in both the offspring and the mother candidate sample. We accepted parentage and full‐ and half‐sibling assignments with a probability > 0.80.

Furthermore, we compared relatedness based on the trioml estimator and haplotypes (individually determined, not concluded from matrilines) within all breeding pairs that were assigned with a probability > 0.80.

#### Analyses of reproductive skew

We used the software SKEW CALCULATOR 2003 (Nonacs 2003), which calculates eight indices of reproductive skew (*S*: Reeve and Ratnieks 1993; *S*_*c*_: Keller and Krieger 1996; *S*_*3*_: Pamilo and Crozier 1996; *Q*: Ruzzante et al. 1996; *λ*: Kokko and Lindström 1997; *I*_*δ*_ and *I*_*p*_: Tsuji and Tsuji 1998; *B*: Nonacs 2000). We conducted analyses for males and females based on three cohorts of pups that emerged between 2011 and 2013. For the period these cohorts were born in, we confirmed the presence of the included candidate males and females in the population, based on capture dates and behavioral observations. We based our conclusions on the *B* index and its corresponding confidence intervals and *P*‐values (Nonacs 2000). In contrast to all other indices, *B* is the only one that takes into account differing lengths of time individuals were present in the parent sample, that is, varying number of cohorts (Nonacs 2000, 2003). *B* can range from −1 to +2. When reproductive skew is greater than expected, *B* is positive; when it is randomly distributed, *B* is 0; when it is more evenly distributed than expected, *B* is negative. We calculated skew for each female unit. In contrast, we pooled all males for this analysis because paternities indicated that males also sired offspring in cases in which there was no regular spatial overlap with the respective female, supported by direct observations of single excursions of males from their usual range.

We based skew calculations only on genetic paternities, because it was not possible to infer paternities from copulation events, or other social traits, for example, there is no paternal care. For females, we referred to 18 emergent pups, for which we could assign genetic and/or social maternities. We excluded two units (H and J) because SKEW CALCULATOR 2003 cannot detect reproductive skew in units with less than two pups. We conducted a power analysis implemented in SKEW CALCULATOR 2003 to determine the power of the dataset to detect skew, estimating significance levels based on 10,000 simulations. We based this analysis on the following demographic means: two mature females; three pups within three cohorts per unit; a probability of 0.7 and 0.3 for the first and second potential mother, respectively, based on the average presence during breeding seasons.

For comparison with the available genetic samples, we also calculated the mean percentage of conceiving females (distension of abdomen), and the mean percentage of pups emerging (usually first sighted in less than 2 weeks after birth) from the observed pregnancies based on direct observations of pregnancies available for the cohorts 2012 and 2013.

## Results

### mtDNA haplotypes

We successfully obtained sequences for 84 of the 96 extracted *M. decemlineata* DNA samples (43 males, 33 females, and eight individuals of unknown sex). We identified 10 distinct mtDNA haplotypes from these individuals. All 10 haplotypes were also recovered in a previous phylogeographic study on *M. decemlineata*, which also included 21 samples from Kirindy Forest (van Vuuren et al. 2012). Of 325 bp, 24 sites were polymorphic. Overall nucleotide diversity (*π*) was estimated at 0.02429 ± 0.00079 and the overall haplotype diversity (Hd) was estimated at 0.786 ± 0.021. The parameter theta, *θ*, was 4.798 and the value of Tajima's D was positive, but not significant (*D* = 1.95545, *P *>* *0.05), suggesting no significant deviation from neutrality.

**Figure 2:**
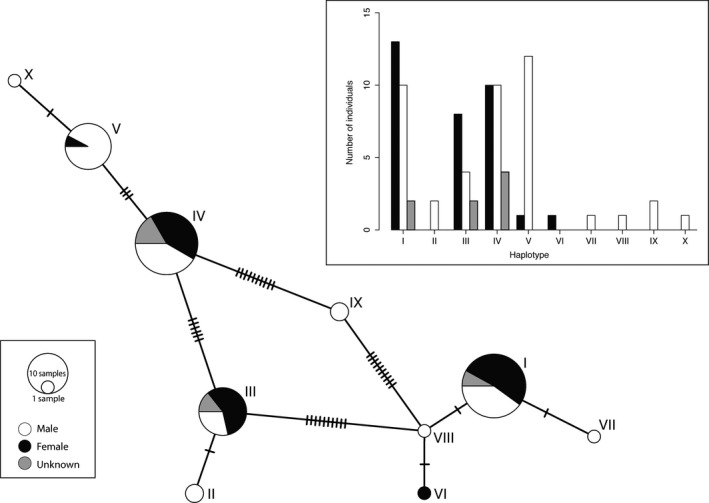
Haplotype network (main pane) and haplotype frequencies (top‐right pane) of male and female narrow‐striped mongooses in Kirindy Forest/CNFEREF. Roman numerals refer to different haplotypes; hatch marks represent the number of mutational changes between haplotypes.

**Figure 3:**
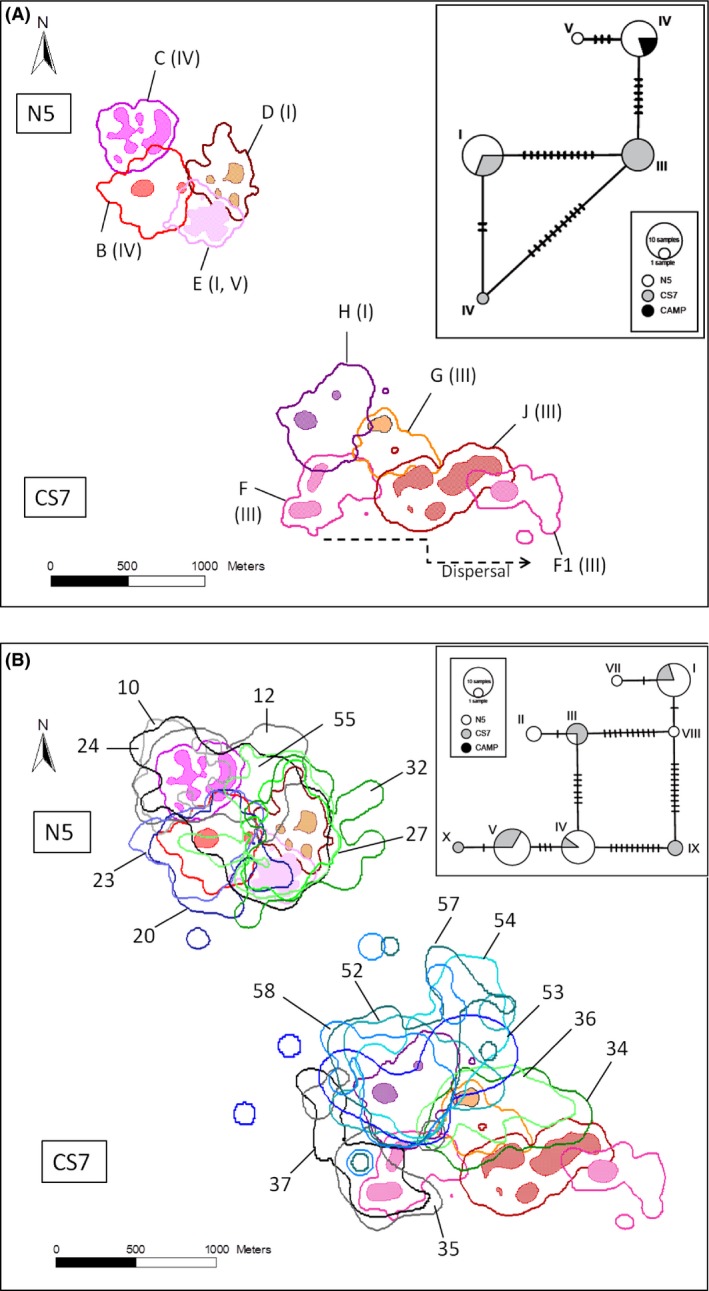
Annual home range and haplotype distributions of nine female units (A, B) and 17 males (B) of narrow‐striped mongooses. Lines represent 90% kernel home ranges; filled areas represent 50% kernel home ranges (=core areas) of females. Letters label female units; numbers label male individuals. Haplotypes are indicated in brackets. N5 and CS7 denote local study areas. The three individuals captured at the site “Camp” were not included in spatial and behavioral data collection.

The frequency distribution of the mtDNA haplotypes was relatively skewed with four haplotypes (I, III, IV, and V) being very common (13–25 individuals) and the remaining haplotypes (II, VI, VII, VIII, IX, and X) occurring at low frequencies (1 or 2 individuals; Fig. 2). Females and males differed strongly in haplotype identity and diversity. Only five different haplotypes were found among females, while nine distinct haplotypes were present among males. While only one haplotype (VI) was unique in females, five haplotypes were exclusively represented in males, suggestive of male migration into the study area. Moreover, haplotype V was found in 12 males but only in one female (Figs. 2, 3). Although there was no significant difference in *π* between the sexes (*π*_♂_ = 0.02423 ±0.00166, *N*_♂_ = 43; *π*_♀_ = 0.02401 ± 0.00098, *N*_♀_ = 33; *P *=* *0.501), haplotype diversity was significantly higher in males (Hd_♂_ = 0.818 ± 0.030, *N*_♂_ = 43; Hd_♀_ = 0.714 ±0.038, *N*_♀_ = 33; *P *<* *0.001). Females in social units tended to share haplotypes. All females of the units A, B, and C displayed haplotype IV; D, E, and H shared haplotype I; F, G, and J displayed haplotype III (Fig. 3A). Female immigration resulted in one unit comprising different haplotypes (Fig. 3A, I and V in unit E). Members of male associations differed in haplotypes. In nine associations, haplotypes of at least two males could be identified. In those nine associations, maximally 50% of males shared the same haplotypes. In 21 of 24 possible dyadic combinations of associated males, haplotypes differed from each other (Table S3).

### Relatedness within sexes, female units, and male associations

The correlation coefficients between observed and expected relatedness values for the seven estimators ranged between 0.745 for ritland (Ritland 1996) and 0.855 for trioml (Wang 2007; Table S4). Hereafter, we describe results only based on trioml, the estimator with the highest correlation coefficient. The estimator trioml is a likelihood method that uses the genotypes of a triad of individuals in estimating pairwise relatedness (*R*). Values of trioml relatedness are constrained to their biologically meaningful ranges [0,1] (Wang 2007). Values for all estimators are reported in Table S4.

**Figure 4:**
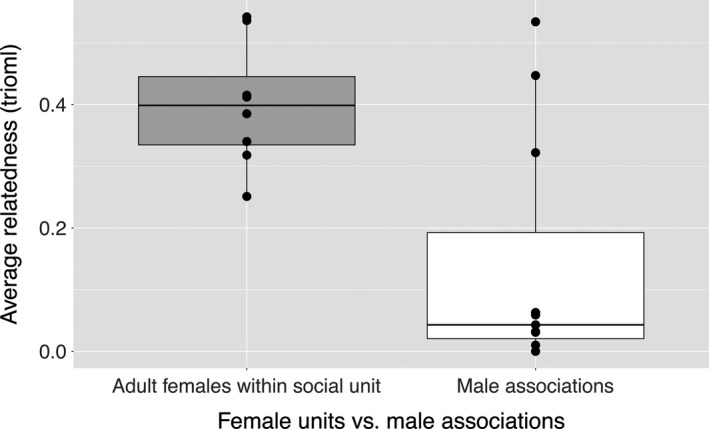
Average relatedness of adult females within social units and males within male associations of narrow‐striped mongooses, based on the trioml estimator.

**Figure 5:**
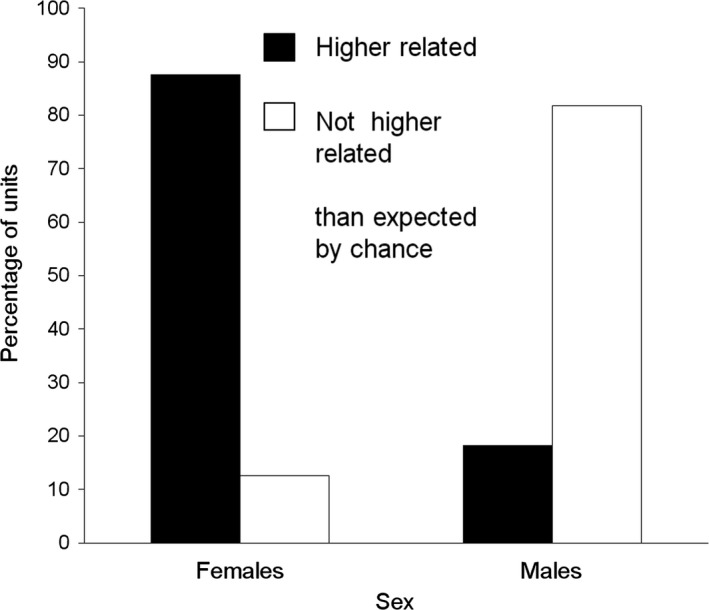
Proportions of sexually segregated social units of female and male narrow‐striped mongooses with regard to the relatedness among adult individuals.

Overall, females in the population were found to be more closely related to each other than males; however, based on trioml estimates, this difference was not significant (*P *>* *0.05). Average pairwise relatedness among adult females belonging to the same social unit was generally higher than expected, with values ranging between 0.251 in unit F and 0.542 in unit C. The only unit in which the relatedness value obtained by trioml was not significantly different from the random distribution was unit G (*R *=* *0.318; *P *=* *0.057; Figs. 4, 5).

Two male associations (2 and 12) had higher relatedness estimates than that of the mean of relatedness estimates based on random permutations, 0.534 (*P *=* *0.023) and 0.447 (*P *=* *0.040), respectively. In the other nine cases, males forming associations were not more closely related to each other than expected by chance. The mean observed relatedness of males within associations ranged between 0 and 0.322 and were not found to be significantly greater than that of randomly generated groups (*P *>* *0.05; Figs. 4, 5).

### Parentages and sibling relationships

Of 22 offspring, paternity and maternity could be assigned to 19 infants and juveniles with a probability > 0.80, thereof 18 with a probability > 0.95 (Table 1). For one juvenile (Md26), none of the candidate fathers were assigned. For two infants, Md51 and Md62, only low probabilities of candidates for parentage, including the observed probable social mothers (Md38 and Md39) were obtained (mothers: 0.486 and 0.163; fathers: 0.293 and 0.025, respectively). For one juvenile (Md50), the adult females of the unit were not assigned as mothers, suggesting that it was presumably offspring of a female previously breeding in this unit, but which had disappeared before the observation period and could not be sampled.

**Table 1:** Parentage assignments for 22 narrow‐striped mongoose offspring sampled from 2011 to 2014

Offspring ID	Sex	Unit ID	Unit size	Cohort	Assigned mother	Probability mother	Assigned father	Probability father	*R* (m/f)
(a) Infants									
Md17	N/A	A	3	2011	Md9	1.0	Md19	0.998	0.103
Md56	N/A	C	4	2012	Md15	1.0	Md12	1.0	0
Md59	N/A	C	4	2013	Md15	1.0	Md12	1.0	0
Md61	N/A	D	3	2013	Md26	0.999	Md27	1.0	0
Md41	F	E	6	2012	Md1	0.965	Md19	0.892	0
Md51	N/A	F	5	2012	(Md38)	(0.486)	(Md19)	(0.293)	(0.077)
Md44	F	G	4	2012	Md48	1.0	Md35	1.0	0
Md62	N/A	G	4	2013	(Md39)	(0.163)	N/A	N/A	N/A
(b) Infants and juveniles									
Md8	F	A	3	2010	Md9	1.0	Md19	1.0	0.103
Md14	F	B	4	2010	Md6	1.0	Md4	1.0	0
Md21	F	C	4	2010	Md15	1.0	Md19	1.0	0
Md22	F	C	4	2011	Md15	1.0	Md19	1.0	0
Md26	F	D	4	2010	Md_MLL11	1.0	N/A		N/A
Md28	M	D	4	2011	Md_MLL11	0.997	Md32	1.0	0.174
Md3	F	E	4	2010	Md1	1.0	Md_MLL9	0.999	0
Md11	M	E	5	2010	Md1	1.0	Md4	1.0	0.386
Md33	F	E	5	2011	Md2	1.0	Md5	1.0	0.079
Md50	F	F	4	2011	(Md63)	(0.749)	Md34	1.0	(0.186)
Md49	M	G	4	2010	Md48	1.0	Md35	1.0	0
Md42	F	H	3	2010	Md45	0.873	Md57	0.967	0.006
Md43	F	H	3	2011	Md45	1.0	Md57	1.0	0.006
Md47	M	J	4	2011	Md46	1.0	Md34	1.0	0

(a): Eight infants born during the observation period of the respective unit; (b): 14 infants and juveniles already present at the beginning of the respective observation period. Parentages with a probability < 0.80 in brackets. *R* (m/f): relatedness within breeding pairs according to the trioml estimator.

Full‐ and half‐sibling relationships are summarized in Tables 2, 3. There were five full‐sibling pairs. Ten half‐sibling pairs shared a common father, while eight pairs shared a common mother. All shared maternities of half‐siblings were within the same social unit, while all shared paternities were distributed among different units.

**Table 2:** Full‐sibling assignments for 10 narrow‐striped mongoose offspring including the cohorts from 2010 to 2013

Full‐sibling IDs	Unit	Full‐sibling probability	Full‐sibling mother	Full‐sibling father	Half‐sibling IDs
Md8, Md17	A	0.998	Md9	Md19	Md21, Md22, *Md41*
Md21, Md22	C	1.0	Md15	Md19	Md8, Md17, *Md41*, Md56, Md59
Md56, Md59	C	1.0	Md15	Md12	Md21, Md22
Md44, Md49	G	1.0	Md48	Md35	–
Md42, Md43	H	0.854	Md45	Md57	–

IDs in italics denote half‐sibship probabilities lower than 0.95.

**Table 3:** Half‐sibling assignments for 14 narrow‐striped mongoose offspring including the cohorts from 2010 to 2013

Half‐sibling IDs	Unit(s)	Half‐sibling probability	Half‐sibling mother(s)	Half‐sibling father(s)	Cohort(s)
Md8, Md21	A, C	1.0	Md9, Md15	Md19	2010
Md8, Md22	A, C	1.0	Md9, Md15	Md19	2010, 2011
Md8, Md41	A, E	0.892	Md9, Md1	Md19	2010, 2012
Md17, Md21	A, C	0.998	Md9, Md15	Md19	2011, 2010
Md17, Md22	A, C	0.998	Md9, Md15	Md19	2011
Md17, Md41	A, E	0.889	Md9, Md1	Md19	2011, 2012
Md14, Md11	B, E	1.0	Md6, Md1	Md4	2010, 2011
Md21, Md56	C, C	1.0	Md15	Md19, Md12	2010, 2012
Md21, Md59	C, C	1.0	Md15	Md19, Md12	2010, 2013
Md21, Md41	C, E	0.892	Md15, Md1	Md19	2010, 2012
Md22, Md56	C, C	1.0	Md15	Md19, Md12	2011, 2012
Md22, Md59	C, C	1.0	Md15	Md19, Md12	2011, 2013
Md22, Md41	C, E	0.892	Md15, Md1	Md19	2011, 2012
Md26, Md28	D, D	0.997	Md_MLL11	N/A, Md32	2010, 2011
Md3, Md11	E, E	1.0	Md1	Md_MLL9, Md4	2010, 2011
Md3, Md41	E, E	0.964	Md1	Md_MLL9, Md19	2010, 2012
Md11, Md41	E, E	0.965	Md1	Md4, Md19	2011, 2012
Md50, Md47	F, J	0.994	N/A, Md46	Md34	2011

Relatedness within breeding pairs (*N *=* *15) was low (mean *R *=* *0.054; range: 0–0.386), with only one pair displaying relatedness comparable to the level within female units (Md1–Md4: *R *=* *0.386; Table 1). For all breeding pairs for which haplotypes could be determined for both parents (*N *=* *7), five pairs differed while two‐ones showed identical haplotypes.

### Reproductive skew

The 22 offspring were fathered by 10 males (i.e., 34.5% of all 29 candidate fathers). Genetic mothers represented 10 of 27 (37.0%) candidate mothers. Indices of reproductive skew are reported in Table 4. *B* skew indices revealed no significant reproductive skew within males or females. 95% confidence intervals (CI) included zero for all units and thus were not significantly different from a random distribution. 95% CIs also overlapped between males and females in both analyses; thus, the sexes were not significantly different in parentage distribution functions (Table 4). However, for two female units (A and C) CIs included maximum *B* values. Thus, monopoly by one female could not be excluded. Our power analysis revealed that 36 units would be necessary to discriminate a skewed distribution from a random distribution, revealing insufficient power of our analysis based on seven units.

**Table 4:** Indices of reproductive skew among 25 male and 17 female narrow‐striped mongooses based on the genetic parentages (in males) and emerged pups (in females) of the cohorts 2011–2013

Units	*N*	*N*_*b*_	*S*	*S*_*c*_	*S*_*3*_	*Q*	*λ*	*I*_*δ*_	*I*_*p*_	*B*	95% CI (*B*)	*P* level (*B*)	*Equal*	*Monopoly*
Males – genetic paternities	25	9	0.65	0.14	0.74	0.03	0.24	1.60	0.24	0.03	−0.07, 0.12	0.066	−0.07	0.90
Females – emerged pups														
A	2	1	1	1	1	1	1	2	0.12	0.06	−0.17, 0.06	0.579	−0.17	0.06
B	3	2	0.33	0.06	0.50	0	0.62	1	0	−0.07	−0.13, 0.49	0.833	−0.13	0.60
C	2	1	1	1	1	1	1	2	0.25	0.04	−0.08, 0.04	0.552	−0.08	0.04
D	3	2	0.33	−0.41	0.50	−0.50	0.62	0	−0.26	−0.13	−0.25, 0.62	1.0	−0.25	0.63
E	3	2	0.41	0.05	0.60	0	0.69	1	0	−0.08	−0.20, 0.35	0.824	−0.20	0.36
F	2	2	0	−1	0	−1	0	0	−1	−0.25	−0.25, 0.24	1.0	−0.25	0.25
G	2	2	0	−1	0	−1	0	0	−1	−0.25	−0.25, 0.24	1.0	−0.25	0.25
Female mean	2.4	1.7	0.44	−0.04	0.51	−0.07	0.56	0.86	−0.27	−0.10		0.958		

*N*: number of individuals; *N*_*b*_: number of individuals that gained at least one parentage within all cohorts; skew indices: *S*: Reeve and Ratnieks (1993); *S*_*c*_: Keller and Krieger (1996); *S*_*3*_: Pamilo and Crozier (1996); *Q*: Ruzzante et al. (1996); *λ*: Kokko and Lindström (1997); *I*_*δ*_ and *I*_*p*_: Tsuji and Tsuji (1998); *B*: Nonacs (2000). *Equal*: minimum *B* value possible (equal sharing of parentages); *Monopoly*: maximum *B* value possible if all parentages are monopolized by the individual with the highest parentage rate.

Based on visual sightings for the cohorts 2012 and 2013, on average 91.7 ± 8.4% (mean ± SD) of mature females (*N *=* *24) conceived, thereof two females twice within the 2013 season. In 78.7 ± 20.0% of observed pregnancies (*N *=* *26), pups emerged. However, from these two cohorts, on average, only 27.7% of pups survived the first year of life (Schneider and Kappeler, in press).

## Discussion

Our study revealed a strong effect of sex on genetic structure. Haplotype diversity was significantly higher in males than in females. Female units were based on matrilines, and some neighboring units showed identical haplotypes. Similarly, relatedness within female units was higher than expected by chance and higher than among males. Members of male associations mostly differed in haplotypes and average pairwise relatedness of associated males was not significantly different from chance. The mating system of *M. decemlineata* is best characterized by mild promiscuity, with potential mate choice but no evidence for reproductive skew in either sex. We discuss these main findings in more detail below.

### Haplotype distribution and relatedness in females

Our finding that almost all females of the same social unit shared a haplotype indicates a matrilinear structure. The presence of identical haplotypes shared among some neighboring units indicates that matrilines exist beyond the level of mother–daughter relationships, and suggests that philopatry is female‐biased. Matrilinearity occurs in several other carnivores, including procyonids, hyaenids, and felids (Waser and Jones 1983; Frank 1986; Packer et al. 1991; Van Horn et al. 2004), and is also prominent among mongooses. In meerkats groups, *Suricata suricatta*, which are mainly composed of a dominant breeding pair and subordinate first‐order relatives, breeding tenure lasts longer for females than for males, and dispersal is male‐biased, while females do not leave the group voluntarily (Clutton‐Brock et al. 2006; Russell et al. 2007; Young et al. 2007). In banded mongooses, historical group fission (“budding”) patterns and philopatry of young result in genetic differentiation, while neighboring groups are not more genetically similar than nonoverlapping groups (Nichols et al. 2012). In the white‐tailed mongoose, *Ichneumia albicauda*, a “solitary” mongoose with strong gregarious tendencies in high‐density populations, matrilinearity of clans was suspected as well (Waser and Waser 1985). Sociality among adult females in *M. decemlineata* is indicated to be more complex than in some solitary herpestids such as the Egyptian mongoose, *Herpestes ichneumon*, and the slender mongooses, *Galerella sanguinea*, where groups do not contain more than one adult female (Rood and Waser 1978; Palomares and Delibes 1993).

In *M. decemlineata*, the haplotype structure among neighboring units and the consistently higher average relatedness among females than males for all estimators suggest that female dispersal is spatially more limited. Similarly, the significant within‐group relatedness of adult females for the large majority of estimators and female units is in accordance with our conclusions based on mtDNA haplotypes and suggests that units are generally formed by mothers and their offspring. However, several estimators found relatedness values not significantly different from a random combination, in particular for two units (F and G), indicating that other mechanisms can influence social organization. Indeed, we observed single cases of dispersal of and immigration by relatively young mature females into units in which breeding positions were vacant after dominant females had died (Schneider and Kappeler, in press). This process resulted in one unit (E) of mixed haplotypes. As in *M. decemlineata*, usually only the pup of the dominant breeding female survives the first year of life, overall fitness benefits for voluntarily dispersing females can be higher when breeding territories are vacant than from staying in their natal group (Clutton‐Brock and Lukas 2012).

### Male relatedness and implications for dispersal and association patterns

The higher haplotype diversity and relatively low relatedness indicate that male *M. decemlineata* disperse further away from their natal unit than females. Male‐biased dispersal has also been observed in solitary and social mongooses (Waser and Waser 1985; Rood 1987; Clutton‐Brock et al. 2002), where the probability of attaining breeding opportunities within the natal group is much lower for males than for females, which presumably drives male bias in dispersal rates (Rood 1987; Cant 2000; Clutton‐Brock et al. 2001b).

In striking contrast to findings reported from other carnivore species, associated male *M. decemlineata* were not more closely related than expected by chance in most cases. In 21 of 24 possible dyadic combinations of associated males, haplotypes differed from each other. Only two of 11 associations revealed significantly higher relatedness than random, based on the trioml estimator. Association 12 included a recently dispersed young male, which joined an immigrant male. Because narrow‐striped mongooses have singleton litters, association partners cannot be littermates. Additionally, we observed disintegration of male associations at the beginning of the mating period and strong variation of their composition across years (Schneider and Kappeler, in press). This pattern differs from the findings on male coalitions ascribed to kin‐directed fitness benefits such as in fossas (Lührs et al. 2013), lions (Bygott et al. 1979; Packer et al. 1991), slender mongooses, and small Indian mongooses, *Herpestes javanicus* (Waser et al. 1994; Hays and Conant 2003). Direct fitness benefits via mutualism, such as reduction in predation risk (Schneider and Kappeler, in press), may therefore explain the formation of male associations in this species.

### Reproductive patterns and implications for the mating system

Based on parentage data, some males fathered offspring of one to two female units in one breeding season, indicating polygynous mating. Females were also observed to mate with males other than the genetic father. In consequence, the mating system is best characterized as mild promiscuity (Schneider and Kappeler, in press). The spatiotemporal distribution of males during the mating season also revealed that males roamed among the ranges of up to three female units instead of monopolizing specific females (T.C.S. and P.M.K., unpubl. data). However, the presence of five full‐sibling pairs across consecutive breeding seasons reflects the effects of either female choice or male monopolization in the general context of promiscuous mating.

Conclusions regarding the level of reproductive skew are difficult to draw due to the small number of units and pups sampled. Accordingly, the power of *B* index calculations was low, not revealing significant skew, although statistically not excluding monopoly by females in some units. Proportions of conceiving females and emerging pups are comparable to low skew societies such as banded mongoose females, where 80% of females conceived and 93% of pregnant females carried to term (Gilchrist et al. 2004). However, from 2012 to 2014, only 28% of emergent narrow‐striped mongoose pups survived the first year of life, all of them born by the dominant female of the unit. The only exception was revealed by genetic maternities of the cohort 2011, when offspring of two females survived, however, born at an interval of several months from each other, and due to a turnover in unit composition which may have coincided with changes in hierarchy relationships. In conclusion, *postpartum* mechanisms are apparently responsible for regulating reproductive success in females, but we have no evidence regarding a possible role of female infanticide, which has been documented in social herpestids (Rasa 1994; Creel and Waser 1997; Gilchrist 2006; Young and Clutton‐Brock 2006; Young et al. 2008).

In contrast to social mongooses, in which one or a few dominant males sire almost all pups (Griffin et al. 2003; Nichols et al. 2010), the potential of male narrow‐striped mongooses to monopolize access to receptive females is limited by two main factors. First, sexual segregation, including female aggression toward males, in combination with large female ranges impede a stable association of males with receptive females (Lukas and Clutton‐Brock 2013). Second, male associations disintegrate at the beginning of the mating period and vary markedly in composition and thus do not provide a reproductive advantage for association partners. In combination with a male‐biased adult sex ratio and the occasional intrusion of outside males into ranges of their conspecifics (Schneider and Kappeler, in press), this apparently creates scramble competition that prevents male monopolization of individual females and therefore high reproductive skew among males.

## Conclusions

This first detailed socio‐genetic investigation in a Malagasy mongoose species revealed similarities to the matrilinear structure found in other carnivores, despite sexual segregation. The lack of high relatedness within male associations indicates that male sociality can arise independent of kin‐directed fitness benefits. Finally, female reproductive competition is also evident in much smaller social units than in group‐living herpestids, despite a formal lack of evidence for significant reproductive skew. The mechanisms causing reproductive skew in females appear to operate mostly postnatally.

## Data Accessibility

MtDNA sequences were uploaded to GenBank and can be accessed through the following accession numbers KU696553–KU696636. Microsatellite genotypes and individual relatedness estimates are available in the Dryad database: doi: 10.5061/dryad.qs777.

## Conflict of Interest

None declared.

## Supporting information


**Table S1.** Primer sequences, repeat motifs and annealing temperatures of the 13 microsatellite loci (Vogler et al. 2009).
**Table S2.** Summary statistics for microsatellite loci including probabilities of deviation from Hardy–Weinberg Equilibrium and null allele frequency estimations.
**Table S3.** Mitochondrial haplotypes of associated individually marked males of narrow‐striped mongooses.
**Table S4.** Relatedness within sexes, female units and male associations of narrow‐striped mongooses, based on seven different relatedness estimators.
**Appendix S1.** Relatedness estimations using the R package related (Pew et al. 2015).
