# Supplementary material for: Genetic population structure and relatedness in the narrow‐striped mongoose (Mungotictis decemlineata), a social Malagasy carnivore with sexual segregation
Source: Ecol Evol. 2016 May 5;6(11):3734–49. doi: 10.1002/ece3.2123 (PMC4864277; doi:10.1002/ece3.2123)
Supplement: Supplementary file 1 — Table S1. Primer sequences, repeat motifs and annealing temperatures of the 13 microsatellite loci (Vogler et al. 2009). Table S2. Summary statistics for microsatellite loci including probabilities of deviation from Hardy–Weinberg Equilibrium and null allele frequency estimations. Table S3. Mitochondrial haplotypes of associated individually marked males of narrow‐striped mongooses. Table S4. Relatedness within sexes, female units and male associations of narrow‐striped mongooses, based on seven different relatedness estimators. Appendix S1. Relatedness estimations using the R package related (Pew et al. 2015). [file ECE3-6-3734-s001.docx]

**Supporting information**

**Journal: *Ecology and Evolution***

**Title: Genetic population structure and relatedness in the narrow-striped mongoose (*Mungotictis decemlineata*), a social Malagasy carnivore with sexual segregation**

**Authors: Tilman C. Schneider^1,2^, Peter M. Kappeler^1,2^ and Luca Pozzi^2^**

^1^Department of Sociobiology/Anthropology, University of Göttingen, Kellnerweg 6, D-37077 Göttingen, Germany

^2^Behavioral Ecology & Sociobiology Unit, German Primate Center, Leibniz Institute for Primate Research, Kellnerweg 4, D-37077 Göttingen, Germany

Correspondance to:

Tilman C. Schneider

E-mail: tilman.carlo@gmail.com

Phone: +49-551-398166

Fax: +49-551-39729

**Table S1.** Primer sequences, repeat motifs and annealing temperatures of the 13 microsatellite loci (Vogler *et al.* 2009).

| Locus ID | Primer sequence 5’-3’ | Repeat motif | Annealing temp. (°C) | Size range |
| --- | --- | --- | --- | --- |
| 41HDZ1 | F: GTCAGCGCAGAACCCAAC  R: ATTCACCCAAGATTGCCC | (CA)_9_TA(CA)_10_–AAACT(CA)_5_ | 58 | 217-244 |
| 41HDZ67 | F: CAGTTTGGCAGTTCCTCAGTAAG  R: TCATTCCTTTTGCGGCTG | (CA)_22_ | 54 | 268-304 |
| 41HDZ71 | F: GGTCCATCCATTCCGTCA  R: CACAAAAACAGTGCGAACCT | (GT)_17_ | 52 | 197-235 |
| 41HDZ78 | F: GTCTGCCAAGTCTGGATGC  R: ATTTTGTGAAGGTCTGATGGG | (CA)_20_ | 58 | 88-131 |
| 41HDZ90 | F: TGTGCCCAATCCATAACCC  R: CACTCCAGACAGCAAACCAAG | (GT)_21_ | 60 | 137-162 |
| 41HDZ105 | F: GGTGTCCGAGAACTCAAGAATA  R: AGCCTGCTATCACATTTACCAA | (CA)_29_ | 54 | 147-196 |
| 41HDZ112 | F: TCACAGAACAAGTTACTCACAAGC  R: TCTCACATTTTCAAAGGACCTC | (CA)_4_CCAA(CA)_5_–  CCAA(CA)_17_ | 54 | 156-192 |
| 41HDZ122 | F: GACACATACACAGTTTCTCTACATCC  R: CTTTGAAGGGAATGACCAGTG | (CA)_19_ | 54 | 112-151 |
| 41HDZ261 | F: TGAATCCATCCCAGGCTG  R: CATCTGAAGGAAGGTTGAAGC | (TG)_5_C(GT)_13_ | 52 | 210-250 |
| 41HDZ592 | F: ACTGTAACATAGTAGGAAATGGATACG  R: TGAGGTTTTTGACTTTTGCTTG | (CA)_15_ | 52 | 170-186 |
| 41HDZ626 | F: GGTTCTGTGTCTCCCTCTCC  R: CTCACTTATCAAACAAAACGGG | (CA)_4_TT(CT)_18_ | 54 | 175-225 |
| 41HDZ633 | F: GGTTATTGCAGGCTATTCTAGGTC  R: CAACTAAATACTCATAAAAAACCAAGC | (CA)_17_ | 52 | 140-183 |
| 41HDZ960 | F: CAACCACTGACTTCTTACTGACAA  R: GGACTTATTTATGTTTAGGAATGTAGAG | (CT)_10_(CA)_14_T(AC)_18_ | 56 | 178-222 |

**Table S2.** Summary statistics for microsatellite loci including probabilities of deviation from Hardy-Weinberg Equilibrium and null allele frequency estimations.

|  |  |  | Homozygotes | | *F_IS_* | | HWE | | | Null allele frequency estimations | | | |
| --- | --- | --- | --- | --- | --- | --- | --- | --- | --- | --- | --- | --- | --- |
| Locus | *k* | *N* | Exp. | Obs. | W&C | R&H | *Chi^2^* | *df* | *P* (exact) | Oo. | Ch. | Br. 1 | Br. 2 |
| 41HDZ1 | 3 | 96 | 52.0 | 54 | 0.051 | 0.087 | 3.887 | 3 | 0.273 | 0.018 | 0.023 | 0.014 | 0.014 |
| 41HDZ67 | 12 | 96 | 15.6 | 10 | -0.064 | -0.047 | 77.770 | 66 | 0.143 | -0.035 | -0.034 | -0.032 | 0 |
| 41HDZ71 | 9 | 85 | 17.5 | 56 | 0.574 | 0.561 | 274.177 | 36 | 0.000 | 0.275 | 0.399 | 0.253 | 0.409 |
| 41HDZ78 | 7 | 96 | 16.9 | 11 | -0.069 | -0.046 | 18.899 | 21 | 0.582 | -0.039 | -0.036 | -0.034 | 0 |
| 41HDZ90 | 6 | 96 | 36.1 | 35 | -0.013 | -0.029 | 9.908 | 15 | 0.724 | -0.001 | -0.009 | -0.007 | 0 |
| 41HDZ105 | 11 | 96 | 19.8 | 34 | 0.192 | 0.201 | 190.283 | 55 | 0.000 | 0.094 | 0.103 | 0.083 | 0.083 |
| 41HDZ112 | 8 | 96 | 31.2 | 32 | 0.018 | 0.027 | 54.321 | 28 | 0.135 | 0.002 | 0.006 | 0.005 | 0.005 |
| 41HDZ122 | 8 | 96 | 21.2 | 17 | -0.051 | -0.048 | 66.764 | 28 | 0.083 | -0.026 | -0.027 | -0.025 | 0 |
| 41HDZ261 | 10 | 96 | 14.7 | 12 | -0.028 | -0.005 | 35.613 | 45 | 0.482 | -0.018 | -0.016 | -0.015 | 0 |
| 41HDZ592 | 6 | 96 | 22.8 | 25 | 0.036 | 0.009 | 11.538 | 15 | 0.607 | 0.017 | 0.016 | 0.013 | 0.013 |
| 41HDZ626 | 8 | 96 | 32.0 | 44 | 0.193 | 0.163 | 35.820 | 28 | 0.030 | 0.089 | 0.104 | 0.075 | 0.075 |
| 41HDZ633 | 10 | 95 | 14.3 | 12 | -0.023 | -0.009 | 48.931 | 45 | 0.117 | -0.017 | -0.014 | -0.013 | 0.031 |
| 41HDZ960 | 11 | 96 | 14.1 | 13 | -0.008 | 0.015 | 48.952 | 55 | 0.384 | -0.009 | -0.007 | -0.006 | 0 |

*k*: number of alleles; *N*: number of individuals; Exp., Obs.: expected and observed number of homozygotes; *F_IS_*: inbreeding coefficient according to Weir & Cockerham (1984), and Robertson & Hill (1984); null allele frequency estimations based on different algorithms: Oo.: Van Oosterhout *et al.* (2004);

Ch.: Chakraborty *et al.* (1992); Br. 1 and Br. 2: Brookfield (1996).

**Table S3.** Mitochondrial haplotypes of associated individually marked males of narrow-striped mongooses.

| Year | Male association ID | Individual ID | Haplotype |
| --- | --- | --- | --- |
| 2012 | 2 | Md19  Md18 | I  V |
| 2013 | 3 | Md12  Md10  Md16  Md18 | II  IV  IV  V |
| 2013 | 4 | Md19  Md24  Md30 | I  IV  V |
| 2013 | 5 | Md27  Md32 | I  ? |
| 2013 | 7 | Md34  Md36 | III  IX |
| 2013 | 8 | Md37  Md35 | III  IX |
| 2014 | 9 | Md27  Md24  Md55 | I  IV  V |
| 2014 | 10 | Md23  Md30  Md20 | V  V  VIII |
| 2014 | 11 | Md58  Md57 | I  X |
| 2014 | 12 | Md34  Md49 | III  ? |
| 2014 | 13 | Md11  Md28  Md24  Md55 | I  I  IV  V |

?: unknown haplotype

**Table S4.** Relatedness within sexes, female units and male associations of narrow-striped mongooses, based on seven different relatedness estimators.

|  |  | *N* | **trioml** | | **wang** | **lynchli** | | **lynchrd** | **ritland** | **quellergt** | **dyadml** |
| --- | --- | --- | --- | --- | --- | --- | --- | --- | --- | --- | --- |
| Correlation coefficients | |  | 0.855 | | 0.846 | 0.837 | | 0.842 | 0.745 | 0.840 | 0.849 |
| *Relatedness within sexes* | | | | | | | | | | | |
| Males | | 23 | 0.068 | | -0.055 | -0.064 | | -0.027 | -0.028 | -0.026 | 0.082 |
| Females | | 24 | 0.088 | | **0.050** | **0.040** | | **-0.011** | **-0.012** | 0.010 | 0.102 |
| *Adult females within social unit* | | | | | | | | | | | |
|  | A | 3 | **0.536** | **0.564** | | | **0.533** | **0.458** | **0.445** | **0.464** | **0.559** |
|  | B | 3 | **0.415** | **0.438** | | | **0.428** | **0.380** | **0.242** | **0.361** | **0.432** |
|  | C | 2 | **0.542** | **0.472** | | | **0.510** | **0.346** | **0.250** | **0.510** | **0.547** |
|  | D | 3 | **0.340** | **0.260** | | | **0.218** | **0.222** | **0.230** | 0.180 | **0.368** |
|  | E | 3 | **0.412** | **0.348** | | | **0.345** | **0.459** | **0.561** | **0.363** | **0.487** |
|  | F | 3 | **0.251** | **0.266** | | | **0.265** | 0.154 | **0.181** | **0.311** | **0.302** |
|  | G | 2 | 0.318 | **0.420** | | | **0.475** | 0.164 | 0.089 | **0.392** | 0.353 |
|  | H | 3 | **0.385** | **0.446** | | | **0.428** | **0.348** | **0.254** | **0.448** | **0.435** |
| *Male associations* | | | | | | | | | | | |
| Year | | | | | | | | | | | |
| 2012 | 2 | 2 | **0.534** | | **0.423** | **0.405** | | **0.396** | **0.171** | **0.416** | **0.536** |
| 2013 | 3 | 4 | 0.059 | | -0.052 | -0.079 | | -0.017 | -0.049 | -0.055 | 0.067 |
|  | 4 | 3 | 0.043 | | -0.152 | -0.155 | | -0.162 | -0.152 | -0.140 | 0.057 |
|  | 5 | 2 | 0.000 | | -0.207 | -0.225 | | -0.104 | -0.047 | -0.140 | 0.000 |
|  | 7 | 2 | 0.000 | | -0.210 | -0.190 | | -0.058 | 0.067 | -0.090 | 0.000 |
|  | 8 | 2 | 0.322 | | 0.295 | 0.335 | | **0.334** | **0.286** | 0.280 | 0.392 |
| 2014 | 9 | 3 | 0.010 | | -0.151 | -0.167 | | -0.061 | -0.061 | -0.094 | 0.015 |
|  | 10 | 3 | 0.031 | | -0.115 | -0.062 | | -0.068 | -0.033 | -0.057 | 0.039 |
|  | 11 | 2 | 0.063 | | 0.157 | 0.055 | | 0.027 | 0.026 | 0.012 | 0.087 |
|  | 12 | 2 | **0.447** | | **0.364** | 0.335 | | **0.385** | **0.454** | **0.356** | **0.461** |
|  | 13 | 4 | 0.032 | | -0.185 | -0.231 | | -0.105 | -0.127 | -0.163 | 0.046 |

*N*: number of individuals; A-H: unit identity; 2-13: identity of male association; in **bold**: significantly higher related than expected by random combination of individuals; negative relatedness values indicate lower relatedness than expected by random combination. Female unit F1 (which derived from unit F) was not included here because only one female was genotyped.

**Appendix S1. Relatedness estimations using the R package *related*** (Pew et al. 2015)

#script to estimate relatedness and compare performance across seven estimators

require(related)

input <- readgenotypedata("full_dataset.txt") #read input file

#Custom Comparisons

simdata <- familysim(input$freqs, 100) # simulation data based on real allele frequencis in the dataset

output <- coancestry(simdata , trioml = 1, wang = 1, lynchli = 1, lynchrd = 1, ritland = 1, quellergt = 1, dyadml = 1) #calculate relatedness using all seven indexes

simrel <- cleanuprvals(output$relatedness, 100)

triomlpo <- simrel[1:100 , 5]

triomlfs <- simrel[(100 + 1) : (2 * 100) , 5]

triomlhs <- simrel[((2 * 100) + 1) : (3 * 100) , 5]

triomlur <- simrel[((3 * 100) + 1) : (4 * 100) , 5]

wangpo <- simrel[1:100 , 6]

wangfs <- simrel[(100 + 1) : (2 * 100) , 6]

wanghs <- simrel[((2 * 100) + 1) : (3 * 100) , 6]

wangur <- simrel[((3 * 100) + 1) : (4 * 100) , 6]

lynchlipo <- simrel[1:100 , 7]

lynchlifs <- simrel[(100 + 1) : (2 * 100) , 7]

lynchlihs <- simrel[((2 * 100) + 1) : (3 * 100) , 7]

lynchliur <- simrel[((3 * 100) + 1) : (4 * 100) , 7]

lynchrdpo <- simrel[1:100 , 8]

lynchrdfs <- simrel[(100 + 1) : (2 * 100) , 8]

lynchrdhs <- simrel[((2 * 100) + 1) : (3 * 100) , 8]

lynchrdur <- simrel[((3 * 100) + 1) : (4 * 100) , 8]

ritlandpo <- simrel[1:100 , 9]

ritlandfs <- simrel[(100 + 1) : (2 * 100) , 9]

ritlandhs <- simrel[((2 * 100) + 1) : (3 * 100) , 9]

ritlandur <- simrel[((3 * 100) + 1) : (4 * 100) , 9]

quellergtpo <- simrel[1:100 , 10]

quellergtfs <- simrel[(100 + 1) : (2 * 100) , 10]

quellergths <- simrel[((2 * 100) + 1) : (3 * 100) , 10]

quellergtur <- simrel[((3 * 100) + 1) : (4 * 100) , 10]

dyadmlpo <- simrel[1:100 , 11]

dyadmlfs <- simrel[(100 + 1) : (2 * 100) , 11]

dyadmlhs <- simrel[((2 * 100) + 1) : (3 * 100) , 11]

dyadmlur <- simrel[((3 * 100) + 1) : (4 * 100) , 11]

trioml <- rep("tri", 100)

wang <- rep("W", 100)

lynchli <- rep("L", 100)

lynchrd <- rep("LR", 100)

ritland <- rep("R", 100)

quellergt <- rep("QG", 100)

dyadml <- rep("di", 100)

estimator2 <- c( trioml , wang , lynchli, lynchrd, ritland, quellergt , dyadml )

Estimator <- rep( estimator2 , 4)

po <- rep("Parent - Offspring", (7 * 100) )

fs <- rep("Full - Sibs", (7 * 100) )

hs <- rep("Half - Sibs", (7 * 100) )

ur <- rep("Unrelated", (7 * 100) )

relationship <- c(po , fs , hs , ur )

relatednesspo <- c( triomlpo , wangpo , lynchlipo, lynchrdpo, ritlandpo, quellergtpo , dyadmlpo )

relatednessfs <- c( triomlfs , wangfs , lynchlifs, lynchrdfs, ritlandfs, quellergtfs , dyadmlfs )

relatednesshs <- c( triomlhs , wanghs , lynchlihs, lynchrdhs, ritlandhs, quellergths , dyadmlhs )

relatednessur <- c( triomlur , wangur , lynchliur, lynchrdur, ritlandur, quellergtur , dyadmlur )

Relatedness_Value <- c( relatednesspo , relatednessfs , relatednesshs , relatednessur )

combineddata <- as.data.frame(cbind(Estimator,relationship,Relatedness_Value))

combineddata$Relatedness_Value <- as.numeric(as.character(combineddata$Relatedness_Value))

#PLOT THE DATA

ggplot(combineddata , aes(x=Estimator, y = Relatedness_Value) , ylim = c(-0.5 , 1.0)) + geom_boxplot() + facet_wrap(~relationship)

urval <- rep(0 , 100)

hsval <- rep(0.25 , 100)

fsval <- rep(0.5 , 100)

poval <- rep(0.5 , 100)

relvals <- c( poval , fsval , hsval , urval)

#estimate correlation value for all the seven indexes

cor( relvals , simrel [ , 5])

cor( relvals , simrel [ , 6])

cor( relvals , simrel [ , 7])

cor( relvals , simrel [ , 8])

cor( relvals , simrel [ , 9])

cor( relvals , simrel [ , 10])

cor( relvals , simrel [ , 11])

#calculate relatedness coefficient for each statistics

reldata <- readgenotypedata("full_dataset.txt") #read input file

grouprel(genotypes = reldata$gdata, estimatorname = "wang", usedgroups = "all",iterations = 1000)

grouprel(genotypes = reldata$gdata, estimatorname = "dyadml", usedgroups = "all",iterations = 1000)

grouprel(genotypes = reldata$gdata, estimatorname = "lynchli", usedgroups = "all",iterations = 1000)

grouprel(genotypes = reldata$gdata, estimatorname = "lynchrd", usedgroups = "all",iterations = 1000)

grouprel(genotypes = reldata$gdata, estimatorname = "ritland", usedgroups = "all",iterations = 1000)

grouprel(genotypes = reldata$gdata, estimatorname = "quellergt", usedgroups = "all",iterations = 1000)

grouprel(genotypes = reldata$gdata, estimatorname = "trioml", usedgroups = "all",iterations = 1000)

]

#estimate relatedness and 95% CI for each dyad

input <- readgenotypedata("full_dataset.txt") #read input file

output <- coancestry(input$gdata, trioml = 2, wang = 2, lynchli = 2, lynchrd = 2, ritland = 2, quellergt = 2, dyadml = 2)

**References**

Brookfield JFY (1996) A simple new method for estimating null allele frequency from heterozygote deficiency. *Molecular Ecology*, **5**, 453-455.

Chakraborty R, De Andrade M, Daiger SP, Budowle B (1992) Apparent heterozygote deficiencies observed in DNA typing data and their implications in forensic applications. *Annals of Human Genetics*, **56**, 45-57.

Pew J, Muir PH, Wang J, Frasier TR (2015) related: an R package for analysing pairwise relatedness from codominant molecular markers. *Molecular Ecology Resources*, **15**, 557-561.

Robertson A, Hill WG (1984) Deviations from Hardy-Weinberg proportions: sampling variances and use in estimation of inbreeding coefficients. *Genetics*, **107**, 703-718.

Van Oosterhout C, Hutchinson WF, Wills DPM, Shipley P (2004) MICRO-CHECKER: software for identifying and correcting genotyping errors in microsatellite data. *Molecular Ecology Notes*, **4**, 535-538.

Vogler B, Bailey C, Shore G*, et al.* (2009) Characterization of 26 microsatellite marker loci in the fossa (*Cryptoprocta ferox*). *Conservation Genetics*, **10**, 1449-1453.

Weir BS, Cockerham CC (1984) Estimating F-statistics for the analysis of population structure. *Evolution*, **38**, 1358-1370.
